# Influence of Comorbidities and Airway Clearance on Mortality and Outcomes of Patients With Severe Bronchiectasis Exacerbations in Taiwan

**DOI:** 10.3389/fmed.2021.812775

**Published:** 2022-01-21

**Authors:** Hung-Yu Huang, Fu-Tsai Chung, Chun-Yu Lin, Chun-Yu Lo, Yu-Tung Huang, Yu-Chen Huang, Yu-Te Lai, Shu-Ting Gan, Po-Chuan Ko, Horng-Chyuan Lin, Kian Fan Chung, Chun-Hua Wang

**Affiliations:** ^1^Department of Thoracic Medicine, Chang Gung Memorial Hospital, Taipei, Taiwan; ^2^College of Medicine, Chang Gung University, Taoyuan, Taiwan; ^3^Department of Thoracic Medicine, New Taipei City Municipal TuCheng Hospital, Chang Gung Medical Foundation, New Taipei City, Taiwan; ^4^Department of Respiratory Care, New Taipei City Municipal TuCheng Hospital, Chang Gung Medical Foundation, New Taipei City, Taiwan; ^5^Center for Big Data Analytics and Statistics, Chang Gung Memorial Hospital, Taoyuan, Taiwan; ^6^Division of Pulmonary and Critical Care, Department of Internal Medicine, Saint Paul's Hospital, Taoyuan, Taiwan; ^7^Biomedical Research Unit, Experimental Studies, National Heart and Lung Institute, Imperial College London, Royal Brompton Hospital, London, United Kingdom

**Keywords:** bronchiectasis, BACI index, severe exacerbation, airway clearance therapy, mortality

## Abstract

Bronchiectasis is characterized by systemic inflammation and multiple comorbidities. This study aimed to investigate the clinical outcomes based on the bronchiectasis etiology comorbidity index (BACI) score in patients hospitalized for severe bronchiectasis exacerbations. We included non-cystic fibrosis patients hospitalized for severe bronchiectasis exacerbations between January 2008 and December 2016 from the Chang Gung Research Database (CGRD) cohort. The main outcome was the 1-year mortality rate after severe exacerbations. We used the Cox regression model to assess the risk factors of 1-year mortality. Of 1,235 patients who were hospitalized for severe bronchiectasis exacerbations, 641 were in the BACI < 6 group and 594 in the BACI ≥ 6 group. The BACI ≥ 6 group had more previous exacerbations and a lower FEV_1_. *Pseudomonas aeruginosa* (19.1%) was the most common bacterium, followed by *Klebsiella pneumoniae* (7.5%). Overall, 11.8% of patients had respiratory failure and the hospital mortality was 3.0%. After discharge, compared to the BACI < 6 group, the BACI ≥ 6 group had a significantly higher cumulative incidence of respiratory failure and mortality in a 1-year follow-up. The risk factors for 1-year mortality in a multivariate analysis include age [hazard ratio (HR) 4.38, *p* = 0.01], being male (HR 4.38, *p* = 0.01), and systemic corticosteroid usage (HR 6.35, *p* = 0.001), while airway clearance therapy (ACT) (HR 0.50, *p* = 0.010) was associated with a lower mortality risk. An increased risk of respiratory failure and mortality in a 1-year follow-up after severe exacerbations was observed in bronchiectasis patients with multimorbidities, particularly older age patients, male patients, and patients with a history of systemic corticosteroid use. ACT could effectively improve the risk for 1-year mortality.

## Introduction

Bronchiectasis is characterized by permanent dilatation of the bronchi and airway inflammation (1), thus leading to the excess mucus secretion that can make the lungs more vulnerable to infection. Infection and exacerbations are associated with respiratory failure and mortality in bronchiectasis (2, 3). Patients with frequent exacerbations, particularly those experiencing three or more exacerbations per year, had worse quality of life, more frequent hospitalizations, and increased mortality over 5 years (4).

The potential risk factors such as hospital admissions and quality of life score have been utilized to predict the risk of death in patients with bronchiectasis (1, 2, 5–7). The Bronchiectasis Severity Index (BSI) scoring system, including physical characteristics, radiological severity, sputum microbiology, dyspnea score, and a history of exacerbation, has been used to predict mortality from bronchiectasis (2). Pneumonia leads to hospitalization and the deterioration of physiological functions in patients with bronchiectasis (8, 9). Respiratory failure is the major cause of death in bronchiectasis (10, 11). Bronchiectasis is characterized by systemic inflammation and multiple comorbidities, thus all-cause related death is higher in patients with bronchiectasis compared to those without bronchiectasis (7, 12). More recently, the bronchiectasis etiology comorbidity index (BACI) scoring system based on 13 comorbidities has been developed and validated to stratify the risk of mortality and hospital admissions in an European cohort (1). We recently used the BACI to assess the severity of bronchiectasis from different etiologies (13), but have not yet analyzed the clinical treatment outcome and mortality of patients with bronchiectasis hospitalized for pneumonia based on BACI scores to stratify their severity.

The long-term accumulation of mucus in the airway of patients with bronchiectasis facilitates bacterial colonization and recurrent lower respiratory tract infection, thus contributing to pneumonia or hospitalization (14). Besides antibiotics, mucoactive drugs and airway clearance therapy (ACT) are also used as an adjuvant treatment of pneumonia in bronchiectasis (15). ACT may facilitate expectoration and improves airway clearance and quality of life (16–18). Although between 40 and 59% of patients with bronchiectasis receive ACT in Europe, the USA, and India (19–21), the role of ACT and mucoactive drugs in improving hospitalization outcomes or in reducing the risk of exacerbation-related readmission in bronchiectasis is still unknown (22).

Although the BACI score has been shown to be a predictor of the risk for mortality (1), it is not known whether this index could be used to stratify disease severity in clinical trials or database studies. In this study, we used the BACI to stratify disease severity and to investigate the factors that may influence the mortality of patients hospitalized for severe exacerbations in a bronchiectasis cohort in Taiwan.

## Methods

### Data Source

In this study, we used the Chang Gung Research Database (CGRD) to construct a multi-institutional bronchiectasis cohort. The CGRD provides the electronic medical records collection from the Chang Gung Memorial Hospital system. The CGRD includes 6.1% of outpatients and 10.2% of hospitalized patients in Taiwan (23). The locations of Chang Gung Memorial Hospital system (three medical centers including Linkou, Taipei, and Kaohsiung branches) and four regional hospitals (Taoyuan, Chiayi, Keelung, and Yunlin branches) and more detailed information about CGRD has been earlier reported (23, 24). The Institutional Review Board of Chang Gung Memorial Hospital approved this study (IRB number: 201800712B0C502).

### Bronchiectasis Cohort

This cohort included adult patients (age ≥ 18 years) with diagnoses of bronchiectasis in the CGRD between January 2008 and December 2016. Patients with at least two bronchiectasis diagnoses [International Classification of Diseases, 9th Clinical Modification (ICD-9-CM) 494.0 or 494.1, or 10th Revision (ICD-10) J47)] in outpatient visits or one diagnosis from the hospitalization record were collected in the cohort. The inclusion criteria of this study were patients with severe exacerbations, which were defined as emergency room (ER) visits or hospitalizations for bronchiectasis-related infective exacerbations (25). Infective exacerbations were defined as the requirement for antibiotics for deterioration in respiratory symptoms (26), and pneumonia was defined by ICD-9-CM codes (481, 482, 483, 485, and 486) with antibiotic use more than 1 week as described earlier (27–29). The diagnosis of bronchiectasis for our enrolled subjects was made by clinical symptoms, history, and the image study of high-resolution CT (HRCT) which was confirmed by a radiologist and a pulmonary specialist. In the cohort, 70% of patients with bronchiectasis from 2002 to 2008 and 90% of patients with bronchiectasis from 2009 to 2016 had a chest CT scan. Therefore, a portion of bronchiectasis diagnosis was based on a clinical history and chest x-ray. About 1,204 patients with bronchiectasis by the ICD code who did not undergo HRCT were excluded. The BACI score was calculated for each subject based on comorbidities obtained from CGRD diagnoses (ICD-9-CM and ICD-10) [1]. Therefore, patients with bronchiectasis were divided into the BACI ≥ 6 group (high risk) and the BACI < 6 group (low and intermediate risk) as previously described (1).

### Outcomes

The main outcomes were the rates of respiratory failure and mortality at 1 year after severe exacerbations. The secondary outcome was in-hospital mortality. Acute respiratory failure (ICD-9-CM: 518.81, ICD-10: J96.0) was defined as the acute onset of respiratory failure during hospitalization with the need for bi-level positive airway pressure or invasive mechanical ventilator use (30). Mild exacerbation was defined as the requirement for antibiotics in a clinic for the deterioration in respiratory symptoms (26).

### Clinical Measurements

The CGRD included inpatient and outpatient clinical data. We retrieved demographic data, image, and microbiology and pulmonary function reports. The total number of affected lobes was identified in CT reports (<2 lobes affected, 2 lobes, or ≥3 lobes affected, lingual lobe as a separate lobe) (5). We collected sputum microbiology reports during hospitalization and within 1 year after hospitalization. Forced expiratory volume in 1 s (FEV_1_) and forced vital capacity <80% predicted value were retrieved from a pulmonary function test, which was performed with a spirometer according to the American Thoracic Society and the European Respiratory Society criteria (31). For etiologies of bronchiectasis patients, as previously described (13), clinicians mostly would review the history of pulmonary TB and pneumonia, and the sputum culture for infection workup was performed, and the possible comorbidities, such as asthma, COPD, and GERD, were evaluated. The COPD was defined by one inpatient or two outpatient codes (491, 492, 496, and 493.2). Clinicians might take an immune or autoimmune survey if the signs of immunodeficiency and connective tissue diseases were present. When primary ciliary dyskinesia was suspected, nasal mucociliary clearance was measured by using the saccharin test. A1-Antitrypsin was evaluated when HRCT revealed the presence of emphysema affecting the lower lobes. Sweat tests were requested if the signs and symptoms suggestive of cystic fibrosis were present.

Medical treatment included antibiotics, systemic corticosteroids, and inhalation medication. ACT was a hospital-based program, including postural drainage and intermittent positive pressure breathing (IPPB), to provide short-term mechanical ventilation *via* a mouthpiece for assistance in clearing mucus from the lungs. The IPPB used was a device of Bird-Mark respirator (Bird Products Corporation, Springs, CA, USA) that delivered the inspiratory pressure at 20–30 cm H_2_O. The frequency of 10–20 times per min was set to maximize the patient's comfort. Both treatments lasted 30 min per session and were given two times daily (morning and late afternoon) during hospitalization. A physiotherapist or respiratory therapists supervised and trained the patients to complete the treatment session. After discharge, the ACT treatment of an IPPB device and postural drainage were conducted once and 1 h each per week in the outpatient clinic. All therapies had medical records. Postural drainage was performed at least four times per week and lasted at least 60 min per session at home. The physiotherapist would call out to remind the patients. The CGRD database is based on real-world clinical practice, and clinicians prescribed ACT for patients with bronchiectasis according to clinical symptoms. The duration of ACT in a clinic was dependent on patients' consent and physicians' prescription.

### Statistical Analysis

Descriptive statistics (mean and SD) were calculated for all continuous variables. Independent *t*-test and χ2 test were used to compare the baseline and main outcome difference of two study groups. A log-rank test was used to compare the survival between the two BACI groups. Univariate and multivariate Cox regression models were used to identify the independent factors that were associated with 1-year mortality and respiratory failure. The analysis was done with the SAS version software; *p* < 0.05 was considered to be statistically significant.

## Results

**Figure 1:**
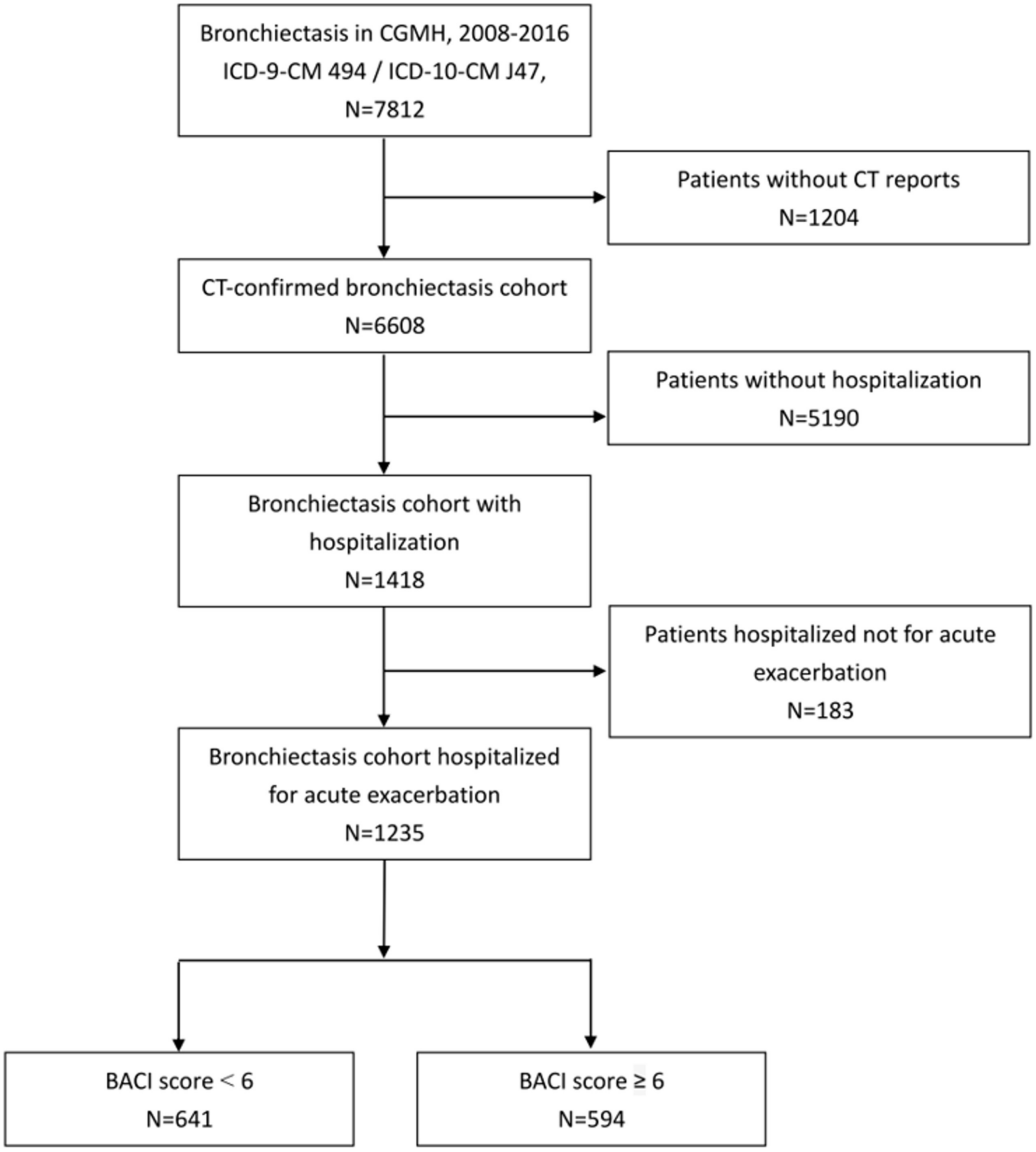
Flow diagram of enrolled subjects.

A total of 7,812 adult patients who had at least two ICD claims (ICD-9-CM 494.0 or 494.1) in outpatient visits or any one ICD claim from hospitalization were included in our bronchiectasis cohort from 2008 to 2016. We excluded the patients without HRCT reports (*n* = 1,204) and those without hospitalization (*n* = 5,190) (Figure 1). A total of 1,235 patients with bronchiectasis hospitalized for pneumonia were enrolled (641 in the BACI < 6 group and 594 in the BACI ≥ 6 group).

The characteristics of the hospitalized patients with bronchiectasis are summarized in Table 1. As compared to the BACI < 6 group, the BACI ≥ 6 group include older age, more male subjects, a lower FEV_1_ at baseline, and a higher frequency of acute exacerbations and hospitalizations in the previous year (Table 1). Of 110 patients who have used macrolide, 94 of them have used it for <1 month and none used macrolide more than 6 months before hospitalization. After discharge, 121 of 177 patients used macrolide for <1 month and only 14 used macrolide as Non-TB mycobacteria (NTM) treatment for more than 6 months. The distribution of macrolide usage in the BACI ≥ 6 and BACI < 6 groups was similar. The airway microbiological status of previous year is listed in Supplementary Table 1. Thus, bronchiectasis patients with a higher BACI score not only manifested more severe comorbidities but also had a worse baseline clinical status. During hospitalization, 857 patients had sputum cultures. *Pseudomonas aeruginosa* (19.1%) was the most common bacterium, followed by *Klebsiella pneumoniae* (7.5%) and *Haemophilus influenzae* (5.5%). In the BACI ≥ 6 group, the rate of *P. aeruginosa* infection was high. A higher proportion of patients in the BACI ≥ 6 group received ACT during hospitalization than those in the BACI < 6 group (52.9 vs. 45.8%, *p* = 0.014). In addition, a greater proportion of patients with bronchiectasis in the BACI ≥ 6 group received ACT for longer than 7 days during hospitalization compared to those in the BACI < 6 group (14.8 vs. 8.9%, *p* = 0.043). Patients in the BACI ≥ 6 group took more systemic corticosteroids than those in the BACI < 6 group (50.7 vs. 29.6%, 9.7 ± 10.6 vs. 6.9 ± 8.4 days, *p* < 0.001). The hospital stays were shorter (11.8 ± 9.3 days) for bronchiectasis patients in the BACI < 6 group than those in the BACI ≥ 6 group (13.8 ± 11.1 days, *p* = 0.001). The two groups exhibited the same in-hospital respiratory failure and mortality rate (Table 2).

**Table 1:** Baseline characteristics.

	**All**	**BACI < 6**	**BACI ≥ 6**	
	***N* = 1,235**	***N* = 641**	***N* = 594**	***P*-value**
Age	67.5 ± 14.4	64.8 ± 15.4	70.5 ± 12.6	<0.001
Sex (female)	633 (51.3%)	365 (56.9%)	268 (45.1%)	<0.001
Etiology				
Post infection (TB)	117 (9.5%)	47 (7.3%)	70 (11.8%)	0.008
Post infection (other)	501 (40.6%)	198 (30.9%)	303 (51.01%)	<0.001
Immunodeficiencies	29 (2.4%)	8 (1.3%)	21 (3.5%)	0.008
Previous AE (mild and severe)	1.9 ± 1.7	1.6 ± 1.6	2.2 ± 1.8	0.002
Hospitalization in previous one year (times/year)	1.4 ± 0.8	1.2 ± 0.6	1.5 ± 0.9	0.001
BACI index	6.8 ± 5.8	2.5 ± 2.1	11.4 ± 4.7	<0.001
Comorbidity				
Malignancy	102 (8.3%)	0 (0.0%)	102 (17.2%)	<0.001
COPD	582 (47.1%)	156 (24.3%)	426 (71.7%)	<0.001
Cognitive impairment	78 (6.3%)	10 (1.6%)	68 (11.5%)	<0.001
Inflammatory bowel disease	8 (0.7%)	2 (0.3%)	6 (1.0%)	0.127
Liver disease	198 (16.0%)	35 (5.5%)	163 (27.4%)	<0.001
Connective tissue disease	56 (4.5%)	10 (1.6%)	46 (7.7%)	<0.001
Iron deficiency anemia	105 (8.5%)	20 (3.1%)	85 (14.3%)	<0.001
Diabetes	282 (22.8%)	59 (9.2%)	223 (37.5%)	<0.001
Asthma	383 (31.0%)	78 (12.2%)	305 (51.4%)	<0.001
Pulmonary hypertension	49 (3.9%)	4 (0.6%)	45 (7.6%)	<0.001
Peripheral vascular disease	16 (1.3%)	2 (0.3%)	14 (2.4%)	0.002
Ischemic heart disease	182 (14.7%)	41 (6.4%)	141 (23.7%)	<0.001
Radiological severity				0.127
<2 lobes	644 (52.2%)	331 (51.6%)	313 (52.7%)	
2 lobes	345 (27.9%)	169 (26.4%)	176 (29.6%)	
>2 lobes	246 (19.9%)	141 (22.0%)	105 (17.7%)	
Lung function				0.004
FEV_1_ <50%	248 (20.1%)	100 (15.6%)	148 (24.9%)	
FEV_1_: 50–80%	255 (20.7%)	120 (18.7%)	135 (22.7%)	
FEV_1_ >80%	280 (22.7%)	157 (24.5%)	123 (20.7%)	
FVC <80%	276 (22.4%)	138 (21.5%)	138 (23.2%)	
Macrolide maintenance				
1 year previous to hospitalization				0.546
None	1,125 (91.1%)	581 (90.6%)	544 (91.6%)	
< one month	94 (7.6%)	53 (8.3%)	41 (6.9%)	
One to six months	16 (1.3%)	7 (1.1%)	9 (1.5%)	
Over six months	0 (0.0%)	0 (0.0%)	0 (0.0%)	

*Data are presented as number (percentage). TB, tuberculosis; AE, acute exacerbation; BACI, bronchiectasis etiology comorbidity index; COPD, chronic obstruction pulmonary disease; FEV_1_, forced expiratory volume in 1 s*.

**Table 2:** Main clinical outcomes of hospitalization.

	**All**	**BACI < 6**	**BACI ≥ 6**	***P*-value**
	***N* = 1,235**	***N* = 641**	***N* = 594**	
Sputum culture in ward	857 (69.3%)	440 (68.6%)	417 (70.2%)	0.578
*Pseudomonas aeruginosa*	164 (19.1%)	69 (15.7%)	95 (22.8%)	0.005
*Klebsiella pneumoniae*	64 (7.5%)	32 (7.3%)	32 (7.7%)	0.708
*Haemophilus influenzae*	47 (5.5%)	29 (6.6%)	18 (4.3%)	0.189
*Fungus*	45 (5.3%)	25 (5.7%)	20 (4.8%)	0.655
NTM	35 (4.1%)	18 (4.1%)	17 (4.1%)	0.918
*Staphylococcus aureus*	27 (3.2%)	13 (2.9%)	14 (3.4%)	0.663
MDR-AB	22 (2.6%)	10 (2.3%)	12 (2.9%)	0.668
ACT	608 (49.2%)	294 (45.8%)	314 (52.9%)	0.014
ACT days				0.043
1–3	282 (22.8%)	143 (22.3%)	139 (23.4%)	
4–7	181 (14.7%)	94 (14.7%)	87 (14.6%)	
>7	145 (11.7%)	57 (8.9%)	88 (14.8%)	
In-hospital medication				
Systemic corticosteroid	491 (39.8%)	190 (29.6%)	301 (50.7%)	<0.001
Days of steroid use^*^	8.7 ± 9.9	6.9 ± 8.4	9.7 ± 10.6	0.002
Antibiotic	1,235 (100.0%)	641 (100.0%)	594 (100.0%)	–
Inhalation acetylcysteine	169 (13.7%)	81 (12.6%)	88 (14.8%)	0.265
Inhalation gentamicin	77 (6.2%)	46 (7.2%)	31 (5.2%)	0.155
Ward days	12.7 ± 10.3	11.8 ± 9.3	13.8 ± 11.1	0.001
Respiratory failure	146 (11.8%)	69 (10.8%)	77 (12.9%)	0.251
Invasive MV	74 (5.9%)	41 (6.4%)	33 (5.6%)	0.551
BiPAP	107 (8.7%)	50 (7.8%)	57 (9.6%)	0.267
In-hospital mortality	37 (3.0%)	18 (2.8%)	19 (3.2%)	0.687

*MDR-AB, multidrug-resistant Acinetobacter baumannii; ACT, airway clearance therapy; MV, mechanical ventilation; BiPAP, bi-level positive airway pressure; NTM, non-tuberculosis mycobacteria*.

* *Daily dose of systemic corticosteroid treatment (prednisolone 20 mg)*.

**Figure 2:**
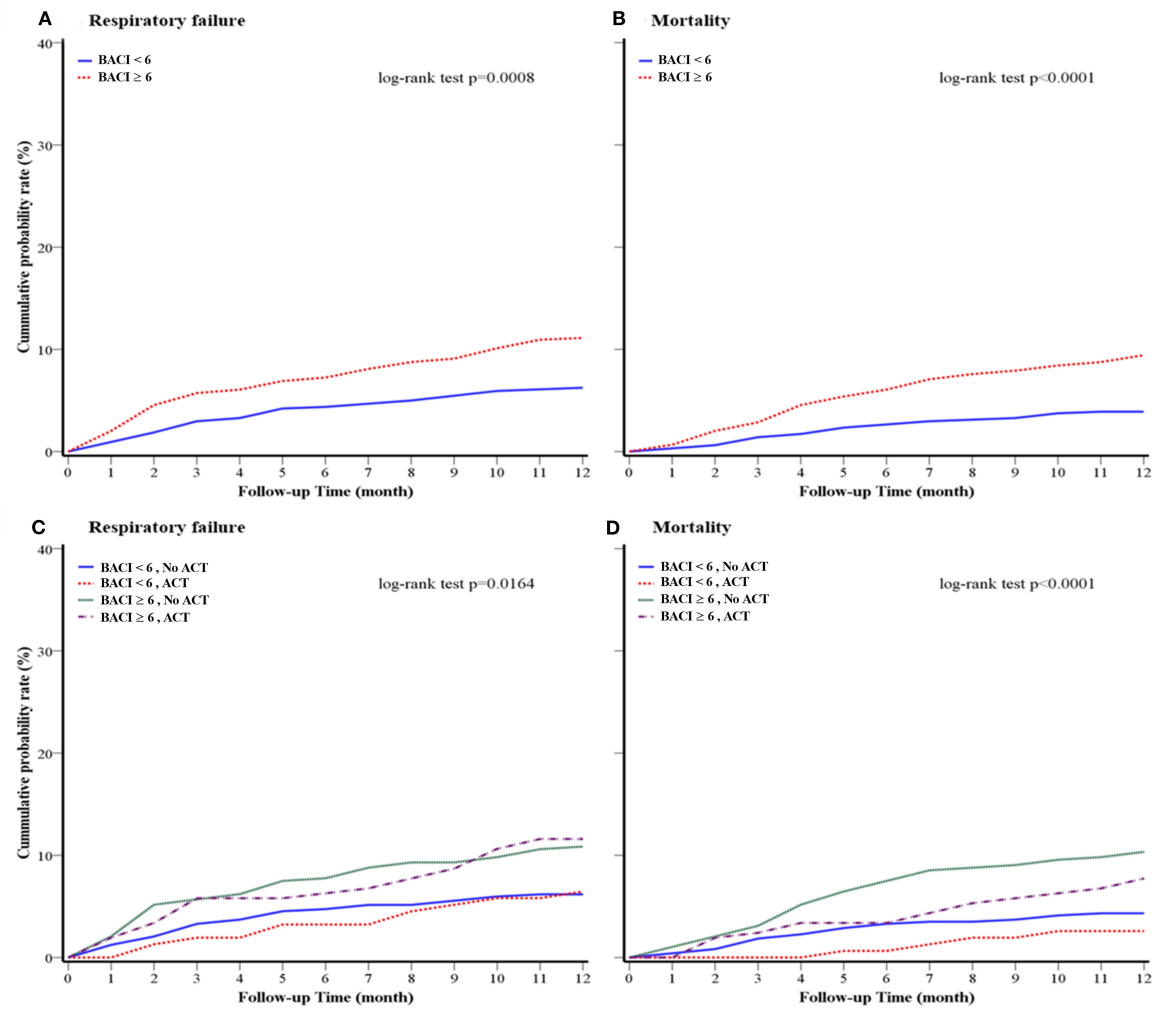
Kaplan–Meier survival curves for **(A)** 1-year respiratory failure and **(B)** 1-year overall mortality of the cohort (BACI groups); **(C)** 1-year respiratory failure and **(D)** 1-year mortality of the cohort (BACI and ACT groups) after a 1-year follow-up. An indicative value of *p* was shown. ACT, airway clearance therapy; BACI, bronchiectasis etiology comorbidity index.

The clinical outcomes of a 1-year follow-up after discharge are shown in Table 3. During the 1-year follow-up period, 669 patients had sputum cultures. NTM (29.3%) was the most common pathogen, followed by *P. aeruginosa* (25.8%), fungus (9.7%), and *K. pneumoniae* (7.9%) in bronchiectasis patients with higher comorbidities. Compared to patients with bronchiectasis in the BACI < 6 group, a higher proportion of patients in the BACI ≥ 6 group received ACT at home during a 1-year follow-up (36.0 vs. 24.9%, *p* = 0.034). During the period of 1-year follow-up, patients with bronchiectasis in the BACI ≥ 6 group took more systemic corticosteroids, antibiotics, and nebulized acetylcysteine. In addition, the rates of acute exacerbation (4.7 vs. 3.9%, *p* = 0.004), respiratory failure (12.0 vs. 6.7%, *p* = 0.002), and 1-year mortality (11.1 vs. 4.2%, *p* < 0.001) were higher in patients with bronchiectasis in the BACI ≥ 6 compared to those in the BACI < 6 group during a 1-year follow-up. The cumulative incidence of respiratory failure and mortality was significantly higher in the BACI ≥ 6 group during a 1-year follow-up (Figures 2A,B). The outcomes of the BACI = 0 group are listed in Supplementary Table 2. The BACI = 0 group (*n* = 248) had the lowest risk of 1-year mortality and respiratory failure (Supplementary Figure 1).

**Table 3:** Main clinical outcomes during a 1-year follow-up.

	**All**	**BACI < 6**	**BACI ≥ 6**	
	***N* = 1,198**	***N* = 623**	***N* = 575**	***P*-value**
Sputum culture	669 (55.8%)	319 (51.2%)	350 (60.8%)	0.001
*Pseudomonas aeruginosa*	173 (25.8%)	61 (19.1%)	112 (32.0%)	<0.001
*Klebsiella pneumoniae*	53 (7.9%)	18 (5.6%)	35 (10.0%)	0.027
*Hemophilia influenzae*	34 (5.1%)	10 (3.1%)	24 (6.9%)	0.022
*Fungus*	65 (9.7%)	34 (10.6%)	31 (8.8%)	0.573
NTM	196 (29.3%)	116 (36.4%)	80 (22.9%)	0.002
*Staphylococcus aureus*	50 (7.5%)	19 (6.0%)	31 (8.9%)	0.118
MDR-AB	35 (5.2%)	13 (4.1%)	22 (6.3%)	0.160
ACT	362 (30.2%)	155 (24.9%)	207 (36.0%)	0.034
ACT duration (months)	4.4 ± 4.4	4.1 ± 4.4	4.5 ± 4.3	0.378
Medication				
Systemic corticosteroid	527 (43.9%)	189 (30.3%)	338 (58.8%)	<0.001
Days of steroid use^*^	93.1 ± 117.8	66.8 ± 101.1	107.8 ± 124.0	<0.001
Antibiotic	857 (71.5%)	392 (62.9%)	465 (80.8%)	<0.001
Inhalation acetylcysteine	153 (12.8%)	49 (7.8%)	104 (18.1%)	<0.001
Inhalation gentamicin	78 (6.5%)	36 (5.8%)	42 (7.3%)	0.285
Macrolide maintenance				0.248
<one month	121 (9.8%)	70 (10.9%)	51 (8.6%)	
One to six months	28 (2.3%)	12 (1.9%)	16 (2.7%)	
Over six months	14 (1.1%)	5 (0.8%)	9 (1.5%)	
AE				
Mild and severe AE	4.4 ± 5.7	3.9 ± 5.8	4.7 ± 5.6	0.004
Severe AE	3.0 ± 3.2	2.5 ± 2.5	3.4 ± 3.6	<0.001
Respiratory failure	111 (9.3%)	42 (6.7%)	69 (12.0%)	0.002
Invasive MV	70 (5.8%)	27 (4.3%)	43 (7.5%)	0.020
BiPAP	76 (6.3%)	24 (3.9%)	52 (9.0%)	0.001
Mortality in 1-year	90 (7.5%)	26 (4.2%)	64 (11.1%)	<0.001

*NTM, non-TB mycobacteria; MDR-AB, multidrug-resistant Acinetobacter baumannii; ACT, airway clearance therapy; AE, acute exacerbation; MV, mechanical ventilation; BiPAP, bi-level positive airway pressure*.

* *Daily dose of systemic corticosteroid treatment (prednisolone 20 mg)*.

The risk factors for increased hospital mortality were older age and multi-drug-resistant *Acinetobacter baumannii* (MDR-AB) infection (age: HR 1.05, *p* = 0.03; MDR-AB: HR 6.36, *p* = 0.01) (Supplementary Table 3 in the *Online supplement*). ACT (HR 0.25, *p* = 0.02) was associated with a lower risk of mortality, while the systemic use of corticosteroids (HR 4.38, *p* = 0.01) was associated with an increased risk of hospital mortality. The risk factors for 1-year mortality with a multivariate analysis included age (HR 4.38, *p* = 0.01), being male (HR 4.38, *p* = 0.01), and systemic corticosteroid use (HR 6.35, *p* = 0.001), while ACT (HR 0.50, *p* = 0.010) could decrease the risk for 1-year mortality (Table 4). The 1-year respiratory failure hazard comparing bronchiectasis with high comorbidities to bronchiectasis with low comorbidities was 2.07 (95% CI: 1.34–5.34) for systemic corticosteroids, 1.69 (95% CI: 1.00–2.83) for nebulized gentamicin, and 0.59 (95% CI: 0.37–0.96) for ACT, which was a low risk for 1-year respiratory failure (Table 5). More importantly, the long-term cumulative incidence of respiratory failure and mortality was significantly reduced by ACT in the BACI ≥ 6 group in a 1-year follow-up (Figures 2C,D). However, the influence of ACT on the one-year respiratory failure and mortality in the BACI < 6 group was not obvious.

**Table 4:** Univariate and multivariate analysis of 1-year mortality.

	**Univariate**			**Multivariate model 1**			**Multivariate model 2**		
	**HR**	**95% CI**	***P***	**HR**	**95% CI**	***P***	**HR**	**95% CI**	***P***
BACI	1.09	1.05–1.11	<0.01	1.03	0.99–1.07	0.142	1.02	0.98–1.05	0.29
Age	1.04	1.02–1.06	<0.01	1.02	1.00–1.04	0.026	1.03	1.01–1.05	0.01
Gender									
Female	1	–	–	1	–	–	1	–	–
Male	2.48	1.57–3.89	<0.01	2.24	1.33–3.76	0.01	2.14	1.32–3.46	0.01
Previous AE	1.21	1.10–1.32	<0.01	0.98	0.87–1.11	0.74	1.01	0.91–1.12	0.82
ACT after discharge	0.85	0.53–1.37	0.51	0.50	0.29–0.87	0.01	0.52	0.31–0.87	0.01
Respiratory failure	1.46	0.83–2.55	0.19				0.93	0.52–1.65	0.79
Sputum infection	1.84	0.99–3.40	0.05						
Specific organisms									
*Pseudomonas aeruginosa*	2.35	1.51–3.66	0.01	0.96	0.56–1.64	0.88			
NTM	0.32	0.17–0.63	0.01	0.46	0.23–0.94	0.03			
MDR-AB	4.48	2.36–8.47	<0.01	1.31	0.57–3.03	0.52			
*Fungus*	2.17	1.22–3.87	0.01	1.82	0.98–3.39	0.06			
*Staphylococcus aureus*	4.54	2.72–7.56	<0.01	1.51	0.83–2.76	0.18			
*Klebsiella pneumoniae*	1.58	0.78–3.16	0.19						
*Hemophilia influenzae*	1.23	0.49–0.051	0.65						
Medical treatment									
Systemic corticosteroid	6.30	3.64–10.84	<0.01	2.67	1.34–5.34	0.01	2.71	1.48–4.96	0.01
Inhalation acetylcysteine	7.85	5.16–11.92	<0.01	1.72	1.01–2.93	0.047	1.84	1.13–3.00	0.02
Inhalation gentamicin	2.46	1.34–4.52	0.01	0.97	0.49–1.90	0.94	0.98	0.52–1.88	0.97

*BACI, bronchiectasis etiology comorbidity index; AE, acute exacerbation; ACT, airway clearance therapy; NTM, non-TB mycobacteria; MDR-AB, multidrug-resistant Acinetobacter baumannii. Model 1 included all significant variables in univariate analysis. Model 2 included significant variables in univariate analysis except specific organisms*.

**Table 5:** Univariate and multivariate analysis of 1-year respiratory failure.

	**Univariate**			**Multivariate model 1**			**Multivariate model 2**		
	**HR**	**95% CI**	***P***	**HR**	**95% CI**	***P***	**HR**	**95% CI**	***P***
BACI	1.06	1.03–1.09	<0.01	1.00	0.96–1.02	0.96	1.00	0.96–1.01	0.44
Age	1.00	0.99–1.01	0.91						
Gender									
Female	1.0	–		1.0			1.0		
Male	1.78	1.21–2.61	<0.01	1.38	0.89–2.13	0.14	1.34	0.90–2.00	0.15
Previous AE	1.17	1.07–1.29	<0.01	0.93	0.82–1.05	0.27	0.97	0.89–1.05	0.38
ACT after discharge	1.18	0.91–1.53	0.22	0.59	0.37–0.96	0.03	0.63	0.41–0.96	0.03
Respiratory failure	1.71	0.99–2.92	0.05						
Sputum infection									
Specific organisms	2.69	1.80–4.00	<0.01	1.40	0.88–2.23	0.16			
*Pseudomonas aeruginosa*	0.45	0.26–0.77	<0.01	0.79	0.45–1.40	0.42			
NTM	3.02	1.57–5.80	<0.01	0.65	0.29–1.44	0.28			
MDR-AB	1.47	0.82–2.63	0.19						
*Fungus*	3.75	2.29–6.14	<0.01	1.48	0.85–2.57	0.16			
*Staphylococcus aureus*	1.19	0.59–2.35	0.62						
*Klebsiella pneumoniae*	1.53	0.71–3.30	0.28						
*Hemophilia influenza*									
Medical treatment									
Systemic corticosteroid	5.85	3.66–9.32	<0.01	2.07	1.13–3.79	0.01	2.52	1.47–4.33	0.01
Inhalation acetylcysteine	7.08	4.87–10.29	<0.01	1.26	0.77–2.05	0.35	1.79	1.13–2.82	0.01
Inhalation gentamicin	4.35	2.75–6.88	<0.01	1.69	1.00–2.83	0.048	1.66	1.02–2.70	0.04

*BACI, bronchiectasis etiology comorbidity index; AE, acute exacerbation; ACT, airway clearance therapy; NTM, non-TB mycobacteria; MDR-AB, multidrug-resistant Acinetobacter baumannii. Model 1 included all significant variables in univariate analysis. Model 2 included significant variables in univariate analysis except specific organisms*.

**Figure 3:**
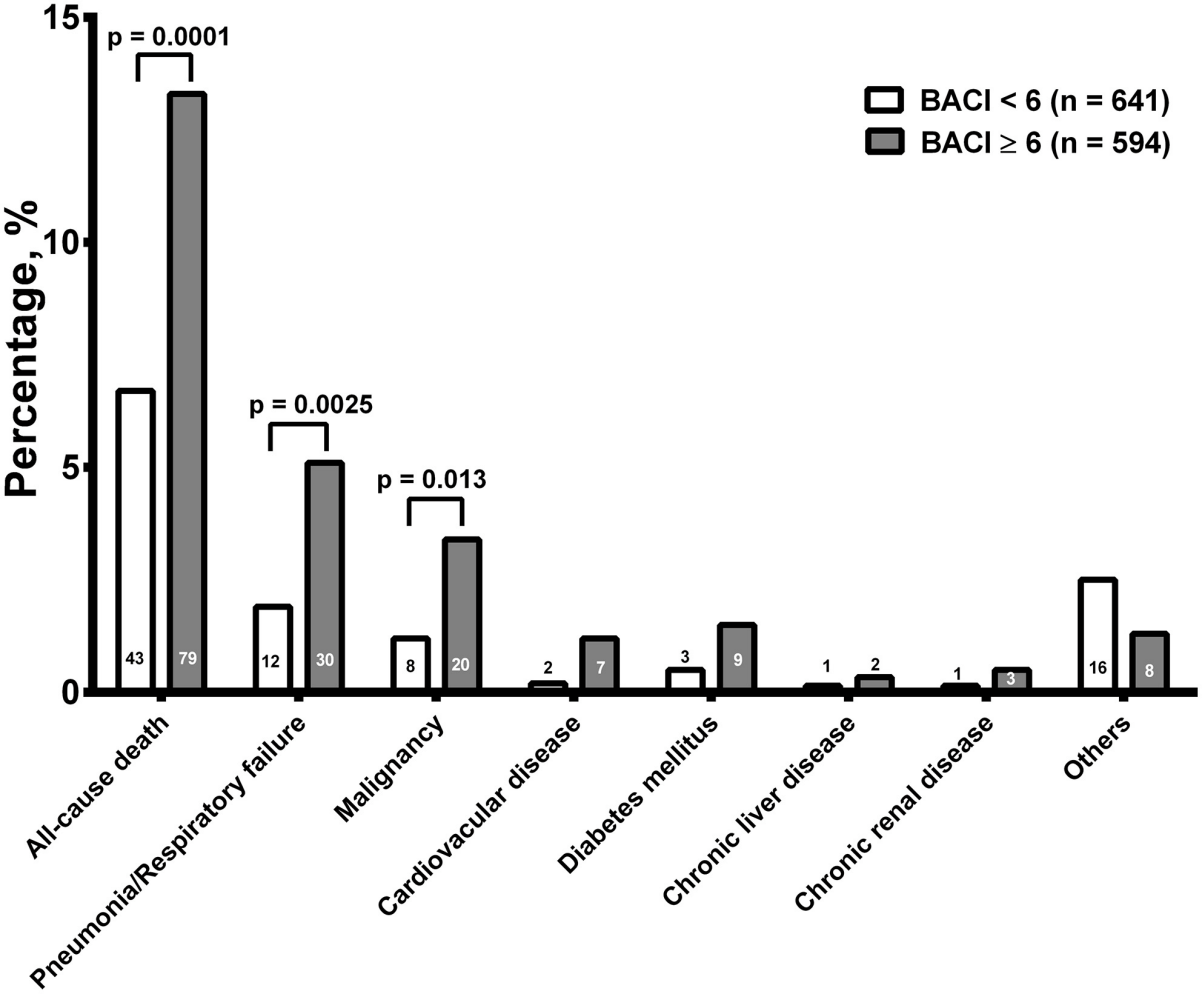
The overall mortality from hospitalization to a 1-year follow-up. The numbers of death and significance are indicated. BACI, bronchiectasis etiology comorbidity index.

The overall mortality from hospitalization to a 1-year follow-up was higher in the BACI ≥ 6 group (13.3%, *n* = 79, *p* = 0.0001) than in the BACI <6 group (*n* = 43, 6.7%, Figure 3). Pneumonia/respiratory failure was the main cause of death, followed by malignancy in both groups. The mortality rates of pneumonia/respiratory failure (5.1% vs 1.9%, *p* = 0.0025) and malignancy (3.4% vs 1.2%, *p* = 0.013) were lower in the BACI < 6 group than in the BACI ≥ 6 group. The existence of the other causes of death, including cardiovascular disease, diabetes, chronic liver disease, and chronic renal disease, showed no difference between the two groups. It seems that pneumonia and higher comorbidities of malignancy may contribute to mortality in the BACI ≥ 6 group.

## Discussion

Our study has used the BACI scoring system to characterize clinical features and comorbidities and to evaluate the risk factor for mortality of patients with bronchiectasis hospitalized for pneumonia in a bronchiectasis cohort in Taiwan. Patients with bronchiectasis in the BACI ≥ 6 group had more previous exacerbations and a worsening of baseline airflow obstruction. Among patients with bronchiectasis, the BACI ≥ 6 group exhibited significantly more frequent acute exacerbations, hospitalizations, and higher rates of respiratory failure and mortality in 1 year after discharge from hospitalization. In the multivariate model, old age, being male, and the use of systemic corticosteroids and nebulized acetylcysteine were independent risk factors for 1-year mortality. More importantly, our results further indicate that ACT can reduce the risk of respiratory failure and mortality during a 1-year follow-up in patients with bronchiectasis hospitalized for pneumonia.

Several indexes such as BSI, FACED, E-FACED, and BACI have been developed, and their scores have been used as the predictors of mortality in bronchiectasis (1, 2, 5, 32). BSI, which incorporates multiple clinical parameters, has become a major tool for evaluating bronchiectasis severity in research studies and clinical trials. Comorbidities have been reported to be associated with an increased risk of mortality in bronchiectasis (1, 12, 33). BACI includes 13 comorbidities that confer a high risk of mortality in bronchiectasis and can be used as a clinical predictive tool independently or with the BSI (10). Thus, our study revealed that patients with high-risk comorbidities (BACI ≥ 6) had a worse baseline physiological state and exhibited significantly more acute exacerbations, hospitalizations, and higher rates of respiratory failure and mortality 1 year after discharge. Those patients hospitalized for pneumonia had a higher incidence of *P. aeruginosa* infection (22.8%). In a 1-year follow-up, we showed that chronic bronchial infection with *P. aeruginosa* was also dominant in bronchiectasis patients with high comorbidities. Notably, bronchiectasis patients with *P. aeruginosa* infection have worse clinical outcomes, such as increased inflammation, greater impairment of the lung function, more exacerbations, and mortality (34, 35). MDR pathogens are difficult to treat, often requiring a combination of antibiotic regimens. A Spanish cohort study reported that MDR pathogens were isolated in 24.5% of patients hospitalized for bronchiectasis exacerbations, and the common pathogens were MDR *P. aeruginosa* and extended-spectrum betalactamase *Enterobacteriaceae* (36). Our results indicated that *P. aeruginosa* and MDR-AB infection are associated with increased mortality in a hospital and a 1-year follow-up in bronchiectasis patients with high comorbidities (Table 4; Supplementary Table 3 in the Online Supplement). Our results would remind physicians to pay increased attention toward a targeted treatment approach to bronchiectasis patients with high comorbidities who were hospitalized for pneumonia with the aim of avoiding respiratory failure and the risk of death.

In our cohort study, NTM infection was the most common pathogen in chronic airway infection of bronchiectasis during a 1-year follow-up after hospitalization. Individuals susceptible of more comorbidities who developed NTM pulmonary disease had increased mortality (37). However, our high comorbidity bronchiectasis with NTM infection was associated with less mortality in a 1-year follow-up after discharge, which is compatible with another report that the bronchiectasis cohort with NTM infection did not show increased mortality relative to the matched control group without NTM infection (12). It might be suggested that the long-term use of macrolide antibiotics, an important drug to treat NTM infection, had a protective effect on the mortality of bronchiectasis patients with NTM infection (38). The number of patients with bronchiectasis and NTM infection in our cohort was too small to draw definite conclusions. There is no evidence-based treatment strategy available for bronchiectasis patients with low or high comorbidities in combination with NTM infection, thus supporting the need for a prospective study to be undertaken. Macrolide maintenance has been reported to prevent acute exacerbation and decrease the frequency of infectious exacerbations using the EMBRACE study and BAT trial (39, 40). Only limited subjects used macrolide for maintenance therapy over 6 months (main usage for NTM treatment). Therefore, the benefit of macrolide maintenance therapy on the outcomes could not be found. A prospective study or the recruitment of more subjects is necessary to evaluate the impact of macrolide maintenance therapy on the outcome of bronchiectasis.

Mucoactive treatments, inhaled antibiotics, and ACT for bronchiectasis are discussed (41). Among bronchiectasis patients with high comorbidities, inhaled mucoactive agents increased the risk of 1-year mortality and respiratory failure and inhalation gentamycin was associated with a worsened 1-year respiratory failure rate, while in those treated with ACT, there was a decrease in the risk of 1-year mortality and respiratory failure. Recommendations for the use of inhaled mucoactive agents in bronchiectasis are indeed weak (15). ACT is beneficial in stable bronchiectasis in improving sputum expectoration, relieving respiratory symptoms, providing a better quality of life, and maintaining the lung function (16–18). Limited studies have investigated the role of ACT in reducing acute exacerbation, hospitalization, or mortality exclusively in patients with non-cystic fibrosis bronchiectasis. In our study, ACT was associated with a reduced risk of in-hospital mortality and of respiratory failure and mortality during a 1-year follow-up in the high-risk comorbidity group (BACI ≥ 6). In contrast, Basavaraj et al. (20) reported that patients with bronchiectasis who used ACTs continuously up to a 1-year follow-up had more frequent chronic infection with *P. aeruginosa*, exacerbations, and hospitalizations and had greater odds for exacerbations in a 1-year follow-up compared with those who did not use ACTs. They found that 58% of patients who used ACTs at baseline did not use ACTs in a 1-year follow-up. In our study, a similar portion of patients with bronchiectasis who used ACTs in hospitalization, but 66% of patients in the BACI ≥ 6 group and 54% of patients in the BACI < 6 group still used ACTs in a 1-year follow-up. It is possible that the higher long-term usage of ACTs in bronchiectasis patients with higher comorbidities may contribute to better clinical outcomes, in terms of mortality and severe exacerbations. There is a particular need to establish that ACTs are effective in which phenotype of patients with bronchiectasis. The modalities of ACT may have a different impact of acute exacerbations on bronchiectasis (16, 42). In our hospital, the ACTs included positioning and gravity-assisted drainage to facilitate sputum removal, and an IPPB device, which may have the effect of augmentation of lung volumes and the prevention of early airway closure during expiration (43), thus helping sputum clearance (44, 45). This means that multiple modalities of ACTs may have different physiological effects on airway clearance and achieve the different clinical outcomes. Currently, there are no similar studies to describe the clinical characteristics or investigate short-term and long-term clinical outcomes of ACT in bronchiectasis with acute exacerbation. Because our study shows the evidence provided by the database, further trials are needed to consolidate the beneficial effects of ACT in exacerbation or in stable bronchiectasis. In addition, nebulized gentamycin may reduce the bacterial burden in the airways and sputum volume, and may decrease neutrophil-related airway inflammation (46, 47). However, our study did not show that inhaled gentamycin had a positive impact on the improvement of 1-year mortality and respiratory failure in bronchiectasis patients with high comorbidities. The role of ACT in combination with mucoactive agents should be investigated in future trials.

Clinically, short-term corticosteroid treatment is often used to control bronchiectasis exacerbations but sometimes is also prescribed to decrease airway inflammation and attenuate the bronchiectasis progression (48). A Cochrane review indicated that corticosteroids may have short-term benefits, but there is lack of evidence for routine use recommendation (49). In our cohort study, bronchiectasis patients with high comorbidities hospitalized for pneumonia and presenting with the use of systemic corticosteroids had a greater risk for mortality (HR: 4.38) in hospitalization as well as mortality (HR: 2.71) and respiratory failure (HR: 2.52) in a 1-year follow-up compared to those with low comorbidities. Corticosteroids may promote the cell immunity dysregulation that could lead to an increase in infections and airway inflammation that could worsen clinical outcomes, such as exacerbations and mortality (50, 51). The long-term use of inhaled corticosteroids has been associated with an increased risk of pneumonia and mortality in adults with chronic obstructive pulmonary disease (52). Our study does not support the widespread use of systemic corticosteroids in bronchiectasis patients with high comorbidities.

Although administrative databases such as the CGRD provide valuable clinical information, they often do not include all the parameters necessary to calculate disease severity scores used in clinical practice. Therefore, the claims-based severity index is developed not to replace the existing clinical scales but rather to provide an alternative tool. In this study, BACI is used to stratify the disease severity. Although CGRD includes demographic information, diagnoses, examinations, laboratory tests, and medications, the clinical scores such as BSI and FACED could not be calculated due to an incomplete record of clinical parameters. Though BACI previously describes the comorbidity of bronchiectasis, our study might provide evidence that BACI could be used to accurately stratify the risk of hospital and 1-year follow-up mortality in CGRD. Future database studies could use the BACI to classify patients with bronchiectasis and evaluate the clinical outcome.

The limitations of this study are as follows. First, because there was no standard protocol of screening etiology or comorbidities in bronchiectasis in CGMH, some comorbidities may be underestimated in the CGRD database. Second, not all patients had sputum culture performed during hospitalization and we did not routinely check sputum culture in the clinic during a 1-year follow-up. Third, ACT included mainly IPPB and postural drainage, so we could not evaluate or compare the outcomes with other ACT regimens such as positive expiratory pressure devices, high-frequency chest wall oscillation, or other modalities. Fourth, because CGRD is a hospital-based database, we could not exactly identify patients previously treated with home oxygen and/or home non-invasive ventilation. Fifth, because this was an observational study from a multi-institution database, treatment selection bias may exist when evaluating the effect of inhalation medicine or ACT in bronchiectasis. Multicenter randomized trials will be needed to better define the optimal regime and duration of treatment (long-term maintenance therapy or repeated short-term therapy), and to compare the effectiveness and safety of different inhaled antibiotics and between inhaled and systemic antibiotic therapies.

In conclusion, BACI stratifies the risk of mortality and respiratory failure in patients with bronchiectasis who admitted for the treatment of pneumonia, making it a good measure of bronchiectasis severity or adjusting for the risk of outcomes in future claims-based studies. ACT reduced the risk of respiratory failure and mortality in a 1-year follow-up. Future trials or database studies are required to validate our observations from this cohort.

## Data Availability Statement

The original contributions presented in the study are included in the article/Supplementary Material, further inquiries can be directed to the corresponding author/s.

## Ethics Statement

The studies involving human participants were reviewed and approved by the Institutional Review Board of Chang Gung Memorial Hospital approved this study (IRB number: 201800712B0C502). Written informed consent is not required for this retrospective database study. Written informed consent for participation was not required for this study in accordance with the national legislation and the institutional requirements.

## Author Contributions

H-YH, H-CL, and C-HW: conceptualization. F-TC and C-YL: investigation. Y-TH, Y-TL, and H-CL: methodology. Y-CH: data curation. Y-TH: validation. H-YH and C-HW: writing—original draft preparation. KC and C-HW: writing—review and editing. All authors contributed to the article and approved the submitted version.

## Funding

This work was supported by Chang Gung Memorial Hospital Research Project Grant (CMRPG3H0931; CMRPG3K2061), Saint Paul's Hospital Research Project (SPMRP-U1-3001), and the Maintenance Project of the Center for Big Data Analytics and Statistics (Grant CLRPG3D0046) at Chang Gung Memorial Hospital.

## Conflict of Interest

The authors declare that the research was conducted in the absence of any commercial or financial relationships that could be construed as a potential conflict of interest.

## Publisher's Note

All claims expressed in this article are solely those of the authors and do not necessarily represent those of their affiliated organizations, or those of the publisher, the editors and the reviewers. Any product that may be evaluated in this article, or claim that may be made by its manufacturer, is not guaranteed or endorsed by the publisher.
