# Supplementary material for: Influence of Comorbidities and Airway Clearance on Mortality and Outcomes of Patients With Severe Bronchiectasis Exacerbations in Taiwan
Source: Front Med (Lausanne). 2022 Jan 21;8:812775. doi: 10.3389/fmed.2021.812775 (PMC8814605; doi:10.3389/fmed.2021.812775)
Supplement: Appendix A — Supplementary data. [file Data_Sheet_1.docx]

**Supplemental Material**

**Influence of comorbidities and airway clearance on mortality and outcomes of patients with bronchiectasis severe exacerbation in Taiwan**

***Hung-Yu Huang,^1,2,3^ Fu-Tsai Chung,^2, 3^ Chun-Yu Lin,^2, 3^ Chun-Yu Lo,^2, 3^ Yu-Tung Huang,^4^ Yu-Chen Huang^2,3^, Yu-Te Lai ^1^, Shu-Ting Gan^4^, Po-Chuan Ko^4^, Horng-Chyuan Lin^2, 3^,* *Kian Fan Chung^5^*, *and Chun-Hua Wang^2, 3^***

*Affiliations*

^1^ *Division of Pulmonary and Critical Care, Department of Internal Medicine,*

*Saint Paul’s Hospital, Taiwan*

*^2^ Department of Thoracic Medicine, Chang Gung Memorial Hospital, Taipei, Taiwan.*

*^3^ College of Medicine, Chang Gung University, Taoyuan, Taiwan*

*^4^ Center for Big Data Analytics and Statistics, Chang Gung Memorial Hospital, Taoyuan, Taiwan.*

*^5^* *Experimental Studies, National Heart & Lung Institute, Imperial College London &*

*Royal Brompton Hospital, London, UK*

**Table S1 Airway microbiology before hospitalization in the previous one year**

|  | **All** | **BACI < 6** | **BACI ≥ 6** |
| --- | --- | --- | --- |
|  | **n=1235** | **n=641** | **n=594** |
| Sputum culture | 363 (29.3%) | 142 (22.2%) | 221 (37.2%) |
| *Pseudomonas aeruginosa* | 101 (27.8%) | 39 (27.5%) | 62 (28.1%) |
| NTM | 86 (23.7%) | 34 (23.9%) | 52 (23.5%) |
| *Klebsiella pneumoniae* | 54 (14.9%) | 19 (13.4%) | 35 (15.8%) |
| *Haemophilus influenzae* | 30 (8.2%) | 12 (8.5%) | 18 (8.1%) |
| *Fungus* | 45 (5.3%) | 11 (7.8%) | 22 (9.9%) |
| *Staphylococcus aureus* | 28 (7.7%) | 10 (7.0%) | 18 (8.1%) |
| Negative | 101 (27.8%) | 4 1(28.9%) | 60 (27.2%) |

Note: NTM, non-tuberculosis mycobacteria

**Table S2 Main clinical outcomes during hospitalization and 1-year follow-up**

| **Hospitalization** | **All** | **BACI = 0** | **1 ≤ BACI < 6** | **BACI ≥ 6** | **p-value** |
| --- | --- | --- | --- | --- | --- |
|  | **n=1235** | **n=248** | **n=393** | **n=594** |  |
| Respiratory failure | 146 (11.8%) | 26 (10.5%) | 43 (10.9%) | 77 (12.9%) | 0.855 |
| Invasive MV | 74 (5.9%) | 18 (7.3%) | 23 (5.9%) | 33 (5.6%) | 0.478 |
| Bipap | 107 (8.7%) | 19 (7.7%) | 31 (7.9%) | 57 (9.6%) | 0.917 |
| Inhospital mortality | 37 (3.0%) | 7 (2.8%) | 11 (2.8%) | 19 (3.2%) | 0.985 |
| **1-year follow-up** | **All** | **BACI = 0** | **1 ≤ BACI < 6** | **BACI ≥ 6** | **p-value** |
|  | **n=1198** | **n=241** | **n=382** | **n=575** |  |
| Respiratory failure | 111 (9.3%) | 8 (3.3%) | 34 (8.9%) | 69 (12.0%) | 0.006 |
| Invasive MV | 70 (5.8%) | 6 (2.5%) | 21 (5.5%) | 43 (7.5%) | 0.072 |
| Bipap | 76 (6.3%) | 3 (1.2%) | 21 (5.5%) | 52 (9.0%) | 0.007 |
| Mortality in 1-year | 90 (7.5%) | 5 (2.1%) | 21 (5.5%) | 64 (11.1%) | 0.037 |

Note: MV, mechanical ventilation; BiPAP: bi-level positive airway pressure

**Figure S1 Kaplan-Meier survival curves for (A) one-year respiratory failure and (B) one-year overall mortality of the cohort (BACI groups)**

**
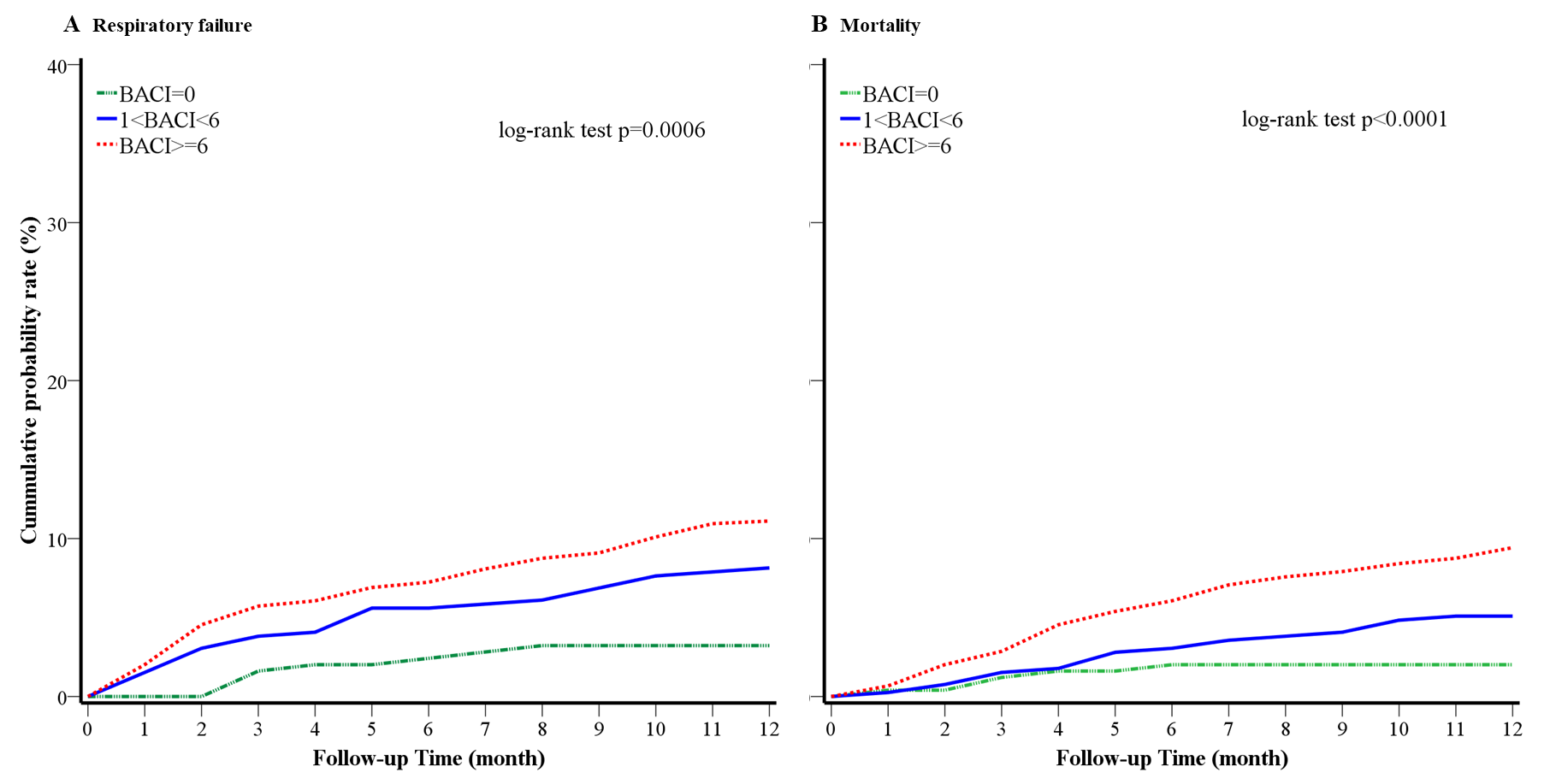
**

**Table S3** **Univariate and multivariate analysis of in-hospital mortality**

|  | **Univariate** | | | **Multivariate**  **Model 1** | | | **Multivariate**  **Model 2** | | |
| --- | --- | --- | --- | --- | --- | --- | --- | --- | --- |
|  | **HR** | **95% CI** | **P** | **HR** | **95% CI** | **P** | **HR** | **95% CI** | **P** |
| BACI | 1.00 | 0.94-1.06 | 0.97 | 0.96 | 0.87-1.07 | 0.46 |  |  |  |
| Age | 1.03 | 1.01-1.05 | 0.03 | 1.05 | 1.01-1.09 | 0.03 | 1.02 | 0.98-1.06 | 0.17 |
| Gender |  |  |  |  |  |  |  |  |  |
| Female | 1 | - | - | 1 |  |  | 1 | - | - |
| Male | 0.85 | 0.44-1.65 | 0.64 | 0.66 | 0.24-1.80 | 0.42 |  |  |  |
| Previous AE | 1.03 | 0.85-1.25 | 0.79 | 0.90 | 0.63-1.31 | 0.59 |  |  |  |
| ACT ward | 0.51 | 0.26-0.98 | 0.04 | 0.25 | 0.08-0.79 | 0.02 | 0.33 | 0.15-0.74 | 0.01 |
| Respiratory failure | 1.84 | 0.92-3.69 | 0.08 | 1.82 | 0.49-6.61 | 0.37 |  |  |  |
| Sputum infection | 1.16 | 0.47-2.85 | 0.75 | 2.08 | 0.50-8.65 | 0.31 |  |  |  |
| \| Specific organisms \| \| --- \| |  |  |  |  |  |  |  |  |  |
| *Pseudomonas aeruginosa* | 1.2 | 0.53-2.71 | 0.66 | 0.51 | 0.13-2.02 | 0.34 |  |  |  |
| NTM | 1.19 | 0.36-4.01 | 0.77 | 0.74 | 0.14-3.96 | 0.72 |  |  |  |
| MDRAB | 0 | 0.00- | 0.99 | 0 | 0.00 | 0.99 |  |  |  |
| *Fungus* | 0.78 | 0.18-3.34 | 0.74 | 0.52 | 0.08-3.11 | 0.46 |  |  |  |
| *Staphylococcus aureus* | 0.57 | 0.08-4.25 | 0.58 | 1.03 | 0.09-12.01 | 0.97 |  |  |  |
| *Klebsiella pneumoniae* | 1.02 | 0.29-3.59 | 0.97 | 0.29 | 0.04-2.21 | 0.23 |  |  |  |
| *Haemophilia influenzae* | 4.21 | 1.70-10.42 | 0.01 | 6.36 | 1.47-27.42 | 0.01 | 3.65 | 1.44-9.26 | 0.01 |
| Medical treatment |  |  |  |  |  |  |  |  |  |
| Systemic corticosteroid | 1.77 | 0.85-3.68 | 0.13 | 4.38 | 1.44-13.28 | 0.01 |  |  |  |
| Inhalation acetylcysteine | 0.54 | 0.22-1.31 | 0.17 | 0.29 | 0.08-1.07 | 0.06 |  |  |  |
| Inhalation gentamicin | 0.70 | 0.22-2.30 | 0.56 | 0.49 | 0.08-3.07 | 0.45 |  |  |  |

Note: BACI, bronchiectasis aetiology comorbidity index; AE, adverse events; ACT, airway clearance therapy; NTM, non-tuberculosis mycobacteria; MDR-AB, multidrug-resistant Acinetobacter baumannii.

Model 1 included all variables in univariate analysis. Model 2 included s all significant variables in univariate analysis.
